# LISA2: Learning Complex Single-Cell Trajectory and Expression Trends

**DOI:** 10.3389/fgene.2021.681206

**Published:** 2021-08-23

**Authors:** Yang Chen, Yuping Zhang, James Y. H. Li, Zhengqing Ouyang

**Affiliations:** ^1^Department of Biostatistics and Epidemiology, School of Public Health and Health Sciences, University of Massachusetts, Amherst, MA, United States; ^2^Department of Statistics, University of Connecticut, Storrs, CT, United States; ^3^Institute for Systems Genomics, University of Connecticut, Storrs, CT, United States; ^4^Department of Genetics and Genome Sciences, School of Medicine, University of Connecticut, Farmington, CT, United States

**Keywords:** scRNA-seq, scATAC-seq, trajectory, pseudo time, development, expression trends

## Abstract

Single-cell transcriptional and epigenomics profiles have been applied in a variety of tissues and diseases for discovering new cell types, differentiation trajectories, and gene regulatory networks. Many methods such as Monocle 2/3, URD, and STREAM have been developed for tree-based trajectory building. Here, we propose a fast and flexible trajectory learning method, LISA2, for single-cell data analysis. This new method has two distinctive features: (1) LISA2 utilizes specified leaves and root to reduce the complexity for building the developmental trajectory, especially for some special cases such as rare cell populations and adjacent terminal cell states; and (2) LISA2 is applicable for both transcriptomics and epigenomics data. LISA2 visualizes complex trajectories using 3D Landmark ISOmetric feature MAPping (L-ISOMAP). We apply LISA2 to simulation and real datasets in cerebellum, diencephalon, and hematopoietic stem cells including both single-cell transcriptomics data and single-cell assay for transposase-accessible chromatin data. LISA2 is efficient in estimating single-cell trajectory and expression trends for different kinds of molecular state of cells.

## Introduction

The fast development of single-cell sequencing technologies has impacted the studies of transcriptomics (Briggs et al., 2018; Farrell et al., 2018; Hochgerner et al., 2018), epigenomics (Rotem et al., 2015; Clark et al., 2018; Gaiti et al., 2019; Sinnamon et al., 2019), proteomics (Palii et al., 2019; Specht et al., 2019), and multiple-omics (Pott, 2017; Bian et al., 2018; Ren et al., 2018; Gu et al., 2019; Liu et al., 2019). The cell number ranges from dozens to millions in single-cell transcriptional applications (Briggs et al., 2018; Farrell et al., 2018; Iacono et al., 2018), and new methods have been developed for all kinds of single-cell data (Butler et al., 2018; Colomé-Tatché and Theis, 2018; Iacono et al., 2018; Liu et al., 2019). Single-cell transcriptomics and epigenomics technologies provide plenty of multi-view data to integrate single-cell RNA sequencing (scRNA-seq) as well as multiple-omics data (Butler et al., 2018; Colomé-Tatché and Theis, 2018; Iacono et al., 2018), identify new cell types/states, discover the relationships of different levels of molecules, build cell trajectories in time and space, and find key regulatory factors in differentiation (Butler et al., 2018; Colomé-Tatché and Theis, 2018; Kulkarni et al., 2019; Luecken and Theis, 2019; Mayr et al., 2019; Tritschler et al., 2019). Here, we focus on computational methods to estimate the cell trajectories from single-cell transcriptomic and epigenomic profiles (Luecken and Theis, 2019).

Many algorithms for estimating cell trajectory have been developed (Ji and Ji, 2016; Liu et al., 2017; Perraudeau et al., 2017; Qiu et al., 2017; Chen et al., 2018; Farrell et al., 2018; Lummertz da Rocha et al., 2018; Street et al., 2018; Cao et al., 2019; Chen H. et al., 2019; Saelens et al., 2019; Setty et al., 2019; Wolf et al., 2019) based on single-cell gene expression data. The main cell trajectory topologies are cycle, linear, tree, and graph (Saelens et al., 2019). Currently, most tree-constructing methods learn trajectory without specifying the root and tips. To date, only slingshot and URD can build global cell trajectories based on user-specified root and tips. URD constructs a branching tree structure based on extended diffusion maps and biased random walks from root to tips (Farrell et al., 2018). Slingshot estimates the global trajectory by minimum spanning tree (MST) and the cell pseudotime by simultaneous principal curves (Perraudeau et al., 2017; Street et al., 2018).

The complexity of single-cell developmental trajectories may come from the following issues. (1) The multiple branches often terminate at different states (Perraudeau et al., 2017; Street et al., 2018). (2) Various development forms include linear, bifurcation, tree, cycle, or disconnected graph (Saelens et al., 2019). (3) Rare cell types are hard to detect, and trajectory-building methods often mix them with other branches (Setty et al., 2019). (4) The trajectory of development is not always linear or irreversible (Mayr et al., 2019). The diversity of cell types/states and complexity of cell differentiation (such as asynchronous or convergent differentiation) can often lead to loop or non-divergent tree structure for single cells. Slingshot and URD can discover the major trajectory but may not work for rare cell populations and converging/diverging branches such as loop structure. Saelens et al. (2019) showed that. Slingshot performs well for more simple trajectories, while PAGA shows higher performance on tree and more complex graph trajectories (Perraudeau et al., 2017; Street et al., 2018; Wolf et al., 2019). In addition, other methods may be suitable for special datasets (Luecken and Theis, 2019). In Slingshot, the principal tree can be applied to any predefined dimension-reduced space and clusters. Users can specify the start cluster and terminal clusters (optional) to construct single or multiple branches. But it tends to find fewer branches than PAGA and Monocle 2 (Qiu et al., 2017), and its scalability is limited (Saelens et al., 2019). PAGA uses graph-like embedding and graph partition to build an abstract graph structure for the cell trajectories with both discrete and continuous cell states (Wolf et al., 2019). Similar to STREAM (Chen H. et al., 2019) and Monocle 2 (Qiu et al., 2017; Cao et al., 2019), PAGA is still overoptimistic for the complexity of cell differentiation. Monocle 2 and STREAM are based on similar tree-building methods by fitting the MST on the dimension-reduced space (Qiu et al., 2017). For Monocle 2, discriminative dimensionality reduction tree (DDRTree) is built directly on the principal component analysis (PCA)/independent component analysis (ICA) space (Qiu et al., 2017; Cao et al., 2019), but Monocle 2 may be affected by noise and thus fail to distinguish correct terminal states for multiple branches (Setty et al., 2019). In STREAM, elastic principal graph is built on modified locally linear embedding (MLLE) space. But the MLLE method is hard to scale to larger datasets. CellRouter is proposed to find dynamic gene expression along a single branch with user-defined source and target, which is not used for estimating global trajectories (Lummertz da Rocha et al., 2018).

To find an improved way to solve non-divergent trajectories, we have developed a fast and flexible trajectory learning method, LISA2, which provides an efficient solution to construct a spanning tree structure by specifying the root and tips. LISA2 builds a *k*-nearest neighbors (kNN) graph from selected principal components and applies a community detection algorithm for clustering. Then, it converts the kNN graph into 3D Landmark ISOmetric feature MAPping (L-ISOMAP) to visualize the cell differentiation in three-dimensional space. By combining the clustering and kNN graph, it can produce a proper spanning tree very fast with specified root and tips. Pseudotime visualization is built on the tree structure by mapping the cells to the tree. To discover interesting gene expression patterns along the cell branch, we use the principal trend analysis (PTA) method (Zhang and Davis, 2013; Zhang and Ouyang, 2018) and identify key gene expression patterns.

Here, we first introduce the workflow of LISA2. Then, we use LISA2 to explore a cerebellum dataset to build a globally convergent cell trajectory (Wizeman et al., 2019). Furthermore, we use LISA2 to build the trajectory of the diencephalon and use PTA to find branch-specific markers. In addition, we apply LISA2 to a single-cell assay for transposase-accessible chromatin (scATAC-seq) dataset from the human hematopoietic [hematopoietic stem cell (HSC)] system to show its potential applications on single-cell epigenome data (Chen H. et al., 2019) we further show the capability of LISA2 in identifying rare cell types. Finally, we compare LISA2 with URD, Monocle 2, and STREAM on the simulation dataset to show the advantages of LISA2 (Papadopoulos et al., 2019).

## Results

### LISA2 Overview

LISA2 is a fast and scalable cell trajectory-building method. Different from LISA (Chen Y. et al., 2019), LISA2 tries to build the tree trajectory using specified root and leaf clusters. LISA2 builds the kNN graph and utilizes community detection methods for clustering based on PCA. LISA2 visualizes scRNA-seq data by two nonlinear dimension reduction methods: UMAP and L-ISOMAP (Silva and Tenenbaum, 2003; McInnes et al., 2018). To build the tree trajectory, users should specify the root and leaf clusters (e.g., based on existing knowledge); we designed a spanning tree to build a tree for the non-leaf cluster, root, and leaf clusters. With the tree trajectory, LISA2 can compute the global pseudotime and use the PTA method (Zhang and Davis, 2013; Zhang and Ouyang, 2018) to discover the main gene expression trends in the specified branch.

**FIGURE 1:**
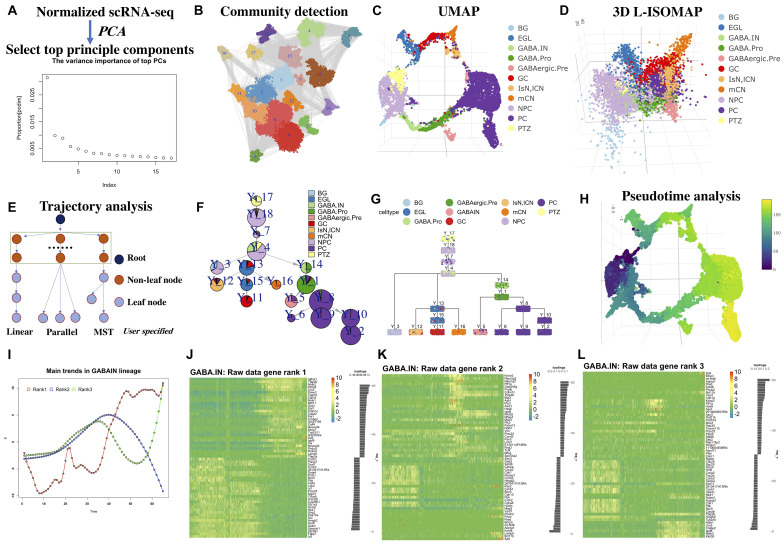
Workflow of LISA2 and cerebellum trajectory analysis. **(A)** PCA and principal component selection. **(B)** Community detection for clustering based on *k*-nearest neighbors (kNN) graph. **(C)** Visualizing in UMAP and **(D)** 3D L-ISOMAP. **(E)** Trajectory building in LISA2 by specifying root and leaf node structure [linear, parallel, and minimum spanning tree (MST)]. **(F)** Trajectory visualization by pie tree plot. **(G)** Trajectory visualization by cell cluster tree plot (each box is a cluster marked by Y_cluster ID, each point represents a cell). **(H)** Pseudotime visualization. **(I)** Gene expression trend analysis by principal trend analysis (PTA) in GABA.IN branch. PTA was applied to the GABA.IN branch to identify main gene expression trends (red, blue, and green) from rank 1 to rank 3. **(J–L)** Heatmap shows gene expression trend of the GABA.IN branch discovered by PTA in ranks 1, 2, and 3. The root cell type PTZ is in yellow in UMAP and 3D-ISOMAP. The leaf cells are divided into three branches. GCs, mCN, and IsN/lCN are derived from EGL. PC is derived from GABAergic.Pre. BG is derived from NPC. We chose linear structure for BG; parallel structure for GC, mCN, and IsN/lCN; tree structure for PC cells. BG, Bergmann glia; EGL, external granule layer cells; GABA.pro, GABAergic progenitor; GABA.pre, GABAergic precursors; GABA.IN, GABAergic interneurons; IsN, isthmic nuclear neuron; mCN, medial cerebellar nuclei; lCN, lateral cerebellar nuclei; NPC, neural progenitor cell; GC, granule cell; MidO, midline organizer cell; PC, Purkinje cell; PTZ, posterior transitory zone.

The main workflow of LISA2 is shown in Figure 1. We used the cerebellum dataset from Wizeman et al. (2019) to demonstrate LISA2. The cerebellum dataset was from mouse embryos at embryonic day (E) 13.5. The raw data were processed using Seurat (Satija et al., 2015), and the cells were annotated based on Wizeman et al. (2019). In total, 9,165 neural cells were used in LISA2 analysis. Non-neural cells were removed when computing the trajectory. Wizeman et al. (2019) identified cell-specific marker genes and subpopulations of cells in E13.5 cerebellum. In Wizeman et al. (2019), Monocle 2 and URD were used to reconstruct several local developmental trajectories for subgroups. However, the global branches of cerebellum development are not accomplished.

Based on the LISA2 method, the top 17 principal components were selected (Figure 1A) for UMAP and community detection (Figure 1B). By checking the clustering results with a range of number of neighbors used in the kNN graph, we finally selected the cluster results with 12 neighbors because the number of clusters is close to the known the number of cell types and the noise level is low (Figure 1C).

The E13.5 cerebellum is composed of three major cell groups: GABAergic neurons, glutamatergic neurons, and neural progenitor cells in the ventricular zone (VZ) (Wizeman et al., 2019). The VZ produces GABAergic neurons and various glia cells. Wizeman et al. (2019) proposed that the posteriormost region of the VZ, referred to as the posterior transitory zone (PTZ), also contains stem cells to sustain the rhombic lip (RL), which gives rise to glutamatergic neurons. Hence, there are three main branches from PTZ to glia, GABAergic, and glutamatergic neurons. The 3D L-ISOMAP plot shows a circle in which medial cerebellar nuclei (mCN), lateral cerebellar nuclei (lCN), and granule cells (GCs) are very close to Purkinje cells (PCs) (Figure 1D). Although they belong to different branches, the global trajectory shows a convergent structure in 3D ISOMAP. This kind of property can be also found in the diencephalon dataset in the following sections.

We built a spanning tree with user-specified root and leaves (Figure 1E). Users can group the leaves into linear, parallel, or MST structure, which will be a substructure in the global trajectory. By comparing the clusters to known cell types, we set cluster 17 as the root; clusters 2, 6, 8, 9, and 10 are PC leaves with MST structure; cluster 5 is GABAergic interneurons (GABA.IN); cluster 3 contains Bergmann glia (BG); cluster 12 is lCN; cluster 16 as mCN; and cluster 11 as GC. Hence, with specified root and leaf nodes, our designed spanning tree can recover a reasonable global tree structure (Figures 1F,G). We designed ways for tree trajectory visualization based on clusters/nodes as shown in Figures 1F,G. In Figure 1F, the pie tree plot shows the tree trajectory and cell type proportion in each node. In Figure 1G, the cell tree plot shows each single cell in each node. Overall, LISA2 successfully builds the global trajectory of early development of the cerebellum.

We then computed the pseudotime of cerebellum development based on the estimated trajectory and visualized it in UMAP space (Figure 1H). The marker genes used in Wizeman et al. (2019) were employed to illustrate gene expression patterns along each branch (Supplementary Figure 1). The heatmap shows dynamic expression changes of transcriptional regulators for all branches and is consistent with the results of Wizeman et al. (2019). For example, in the BG_GABA.pro heatmap in Supplementary Figure 1K, the GABAergic marker genes are mostly expressed in cluster 14 and the marker genes of BG are mostly expressed in cluster 3. The GABAergic neurons include both PCs and GABA.IN, which share common marker genes *Foxp2* and *Gad2*. *Pax2*, *Gad1*, *Pnoc*, and *Glra2* are specifically expressed in GABA.IN. *Tle1* and *Islr2* are expressed in PC cells.

Next, we used LISA2 to discover the main gene expression patterns for all branches. For cerebellum and diencephalon datasets, we used the scaled data from Seurat (v1.4.0) for PTA. We found that the rank 1 trends go up or down for all branches (Supplementary Figure 2). One can find the predominant gene expression trends along the branch and select the driver genes based on the scores of genes that represent the contributions to the trends. In Figure 1I, three different gene expression curves represent the rank 1 to rank 3 trends along the GABA.IN branch. The corresponding heatmaps in Figures 1J–L show cascade gene expression along the GABA.IN branch from ranks 1 to 3. Commonly, three ranks are enough for detecting main gene expression patterns. The trend in rank 1 can show the most prevalent gene expression pattern followed by ranks 2 and 3. The genes are ranked from negative scores to positive scores. A positive or negative score reflects a positive or negative contribution to the trend. For the GABA.IN branch, we found that the GABAergic markers *Lhx1/5* have high scores in ranks 1 and 3 (Supplementary Figure 3D). The marker of GABAergic precursor *Kirrel2* shows the highest positive score 0.15 in rank 2. *Neurog1* also has a high score, 0.13 (Figure 1K and Supplementary Figure 3D). *Sp9*, a regulator of GABAergic neuronal development (Li et al., 2018; Xu et al., 2018; Tao et al., 2019), shows the highest score in the heatmap in rank 3 (Figure 1L).

The PTA scores and rank 1 heatmaps of other branches are also shown in Supplementary Figures 2, 3. The genes discovered by the PTA method are highly or lowly expressed at the ends of the branches. Hence, one can distinguish the branch-specific genes based on the scores from rank 1. We also found that some genes are highly expressed in the middle of the branches, consistent with the ranks 2 and 3 trends (Figure 1K). By PTA analysis of the highly variable genes, we found that the marker genes show high absolute scores in ranks 1–3. Their gene expression trends were captured by PTA.

### Reconstructing Complex Single-Cell Trajectories for the Diencephalon

To further assess the performance of LISA2, we applied LISA2 to scRNA-seq data from the embryonic diencephalon. The embryonic diencephalon plays important roles in the forebrain, which connects the anterior forebrain and the rest of the nervous system (Sherman, 2007; Hikosaka, 2010; Guo and Li, 2019). The scRNA-seq data of the diencephalon are from E12.5 mouse embryos and contain 7,365 filtered cells. Guo and Li (2019) analyzed the spatial origins of the cell groups and the molecular features of the diencephalon region (Guo and Li, 2019). With URD, built a developmental trajectory of the diencephalon with six cell branches and described the developmental cascades. We removed the low-quality and non-neural cells based on the Seurat result of Guo and Li (2019) and obtained 6,952 cells. The scaled data from Seurat were used for trajectory analysis.

**FIGURE 2:**
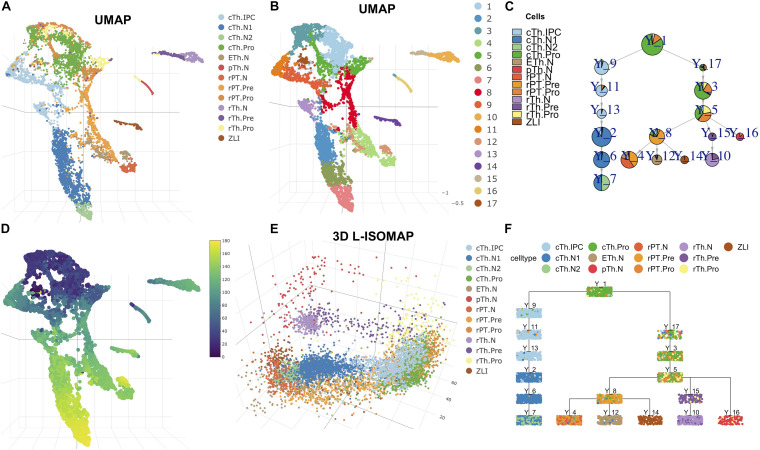
Cell trajectory of mouse diencephalon at E12.5. **(A)** UMAP visualization of cell types. **(B)** Clustering results shown in UMAP. **(C)** Pie tree plot shows the cell types and trajectory. **(D)** The pseudotime distribution in UMAP. **(E)** Diencephalon cell differentiation in 3D L-ISOMAP. **(F)** Cell cluster tree plot shows the differentiation trajectory of the diencephalon (each box is a cluster marked by Y_cluster ID, each point represents a cell). The cell type names are as follows. rTh.pro, rostral thalamic neurons progenitor; rTh.pre, rostral thalamic neurons precursor; rTH.N, rostral thalamic neurons; rPT.Pro, rostral pretectal neural progenitor; rPT.pre, rostral pretectal neural precursor; rPT.N, rostral pretectal neurons; cTh.Pro, caudal thalamic neural progenitor; cTh.IPC, caudal thalamic intermediate progenitor; cTh.N1, caudal thalamus neuron I; cTh.N2, caudal thalamus neuron II; pTh.N, prethalamic neurons; ETh.N, epithalamic neurons; ZLI, zona limitans intrathalamica.

In Figures 2A,B, we showed the cell types and clustering annotation in UMAP space. The clustering results are from Leiden community detection algorithm on kNN graph with *k* = 14. We selected cluster 1 as the root, which contains progenitor cell types of the rostral thalamus (rTh), rostral pretectum (rPT), and caudal thalamus (cTh). Cluster 12 is annotated as epithalamus (ETh). Clusters 2, 6, and 7 are annotated as caudal thalamus neuron (cTh.N). Cluster 4 is annotated as rostral pretectal neurons (rPT.N). Cluster 10 is annotated as rostral thalamic neurons (rTh.N). Cluster 14 is annotated as zona limitans intrathalamica (ZLI). Cluster 16 is annotated as prethalamic neurons (pTh.N). From 3D L-ISOMAP, we found that there were three branches that correspond to cTh, rTh, and rPT. The ETh and rPT branches are derived from the rPT precursors. In 3D L-ISOMAP, cTh and rPT branches are close. cTh.pro and rPT.pro are mixed and separated from rTh.pro cells. ZLI, ETh.N, rPT.N, and cTh.N are close at the end of development (Figure 2E). With LISA2, the trajectory of the diencephalon is shown in Figures 2C,F. cTh branch is separated from other branches. rPT.N, Eth.N, and ZLI share a common parent cluster that contains the rPT.pre cell type. rTh.N and pTh.N share a common parent cluster. The trajectory is consistent with the close relationship of the rTh and pTh and the ETh, and rPT.

We then computed the pseudotime of cells (Figure 2D). We plotted the gene expression along the branch for some transcription factors or marker genes from Guo and Li(2019; Supplementary Figure 4). For example, in cTh branches, temporal expression profiles of gene *Atad2*, *Birc5*, *Id3*, and *Hes1* (cell cycle and apical progenitors), *Neurog1*, *Neurog2*, *Insm1*, and *Cdkn1c* (basal progenitors), and *Gbx2* and *Rora* (postmitotic) are very consistent with those in Guo and Li (2019).

With the pseudotime and scaled data, we applied PTA to discover gene expression patterns in each branch (Supplementary Figure 5). In each branch, we show the gene expression heatmap in rank 1 corresponding to the red trend curve in rank 1 (Supplementary Figure 5). For most marker genes, the absolute PTA scores are high (Supplementary Figure 5). For example, the scores of the *Dlx5/1* genes in rank 1 of the pTh branch are the most negative and ranked at the bottom of the heatmap (Supplementary Figures 4A, 5A, 6A). The *Hmgb2* gene shows a high positive score with its gene expression profile (Supplementary Figure 6A) consistent with the trend in rank 1 of the pTh branch (Supplementary Figure 5A). The *SHH* protein is produced by ZLI to regulate the diencephalon development, but its expression is low and not detected by scRNA-seq. Correspondingly, its PTA scores are zero (Supplementary Figures 4C, 6C).

### Discovering Cell Trajectories in Single-Cell Assay for Transposase-Accessible Chromatin for Hematopoietic Stem Cells

The multi-omics single-cell technologies have the ability to detect chromatin accessibility, in addition to RNA transcription. We applied LISA2 on scATAC-seq data from Chen H. et al. (2019) to demonstrate the ability of LISA2 to analyze single-cell epigenomics data. The scATAC-seq data were preprocessed by STREAM and chromVAR (Schep et al., 2017) from human bone marrow, which contains nine cell types. We used the processed scATAC-seq matrix, which represents the accessibility *z*-scores of cells (2,034) and transcription factor binding motifs (8,192; 7-mers). The principal components used are the same as those used in the STREAM analysis (Chen H. et al., 2019).

**FIGURE 3:**
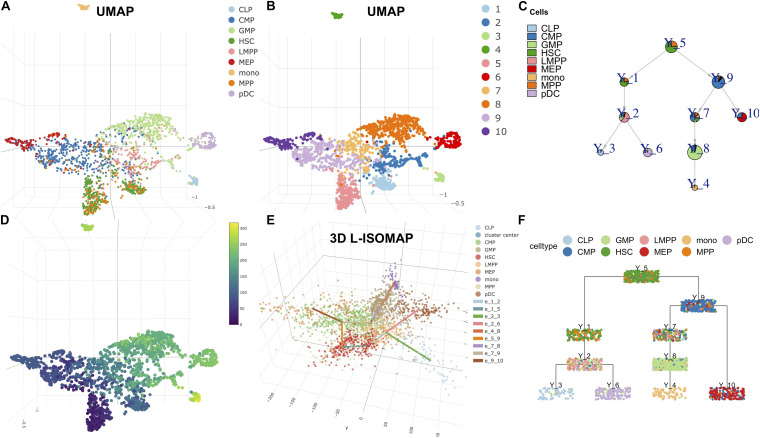
LISA2 analysis of the development of hematopoietic stem cells (HSC) using single-cell sequencing assay for transposase-accessible chromatin (scATAC-seq) data. **(A)** The cell type visualization in UMAP. **(B)** The clustering results on UMAP. **(C)** Pie tree plot shows the cell types and trajectory. **(D)** The pseudotime plot in UMAP. **(E)** The 3D-ISOMAP and trajectory visualization. **(F)** Cell cluster tree plot shows the differentiation trajectory of HSC. All cell types include lymphoid multipotent progenitors (LMPPs), granulocyte monocyte progenitors (GMPs), plasmacytoid dendritic cells (pDCs), lymphoid progenitor cells (CLPs), common myeloid progenitors (CMPs), megakaryocytic cells (MEP), multipotent progenitors (MPPs), and monocytic cells (mono).

In Figures 3A,B, we used UMAP to visualize the cell annotation and clusters. Cell clustering was determined by Louvain community detection algorithm on kNN graph with *k* = 12. HSCs corresponding to clusters 5 and 1 were assigned as the root. Cluster 6 was set as the leaf group of plasmacytoid dendritic cells (pDCs). Cluster 3 was set as the leaf of lymphoid progenitor cells (CLPs). Cluster 4 was set as the leaf of monocytic cells (mono). Cluster 10 was set as the leaf group of multipotent progenitors (MEPs). Hence, we produced the single-cell trajectory (Figures 3C,F) and pseudo time (Figure 3D). From the L-ISOMAP (Figure 3E), we identified four clearly separated non-root branches. The interactive 3D ISOMAP of the HSCs can be seen in the Supplementary Data. The HSCs in red are the root. Based on the trajectory, pDCs and CLPs are close to each other. But MEPs and mono are much further away. The trajectory is consistent with the one obtained by STREAM (Chen H. et al., 2019).

At last, we applied the PTA to the four branches using the *z*-scored scATAC-seq data (Supplementary Figure 7). We identified branch-specific 7-mers in rank 1 for the four branches. For example, the rank 1 score of AGATAAG is −0.049, which contributes to the rank 1 trend along the MEP branch negatively. The rank 1 score of TGTGCAA is −0.047, which contributes to the rank 1 trend in the mono branch negatively. Consistently, the two 7-mer DNA sequences were shown to be mapped to transcription factor motifs of GATA1 and CEBPA respectively, which predominantly regulate the blood development and differentiation to erythroid and myeloid (Chen H. et al., 2019). Their expression data are also shown in Supplementary Figures 7A,B.

### Discovering Rare Cell Lineages

Rare cell types are hard to identify from single-cell profiling data due to small number of cells or low abundance. They may have important functions in development. For example, the Cajal–Retzius cells are important to modulate early cortical patterning and rare choroid plexus that produces cerebrospinal fluid (Griveau et al., 2010; Pollen et al., 2015). There are some existing methods to detect rare cell types from scRNA-seq data. CellSIUS (Wegmann et al., 2019), RaceID3 (Grün et al., 2016), GiniClust2 (Tsoucas and Yuan, 2018) are two-step clustering methods using global clustering first and then doing subclustering to identify rare cell types. scAIDE used autoencoder with multidimensional scaling (MDS) encoder for dimensionality reduction and random projection hashing-based *k*-means clustering to detect rare cell types (Xie et al., 2020). DeMeo and Berger (2021) used Shannon component analysis for dimensionality reduction and assigned an information score to each transcript to define rare cell types. In these methods, dimension reduction and clustering strategies are important for rare cell type detection.

**FIGURE 4:**
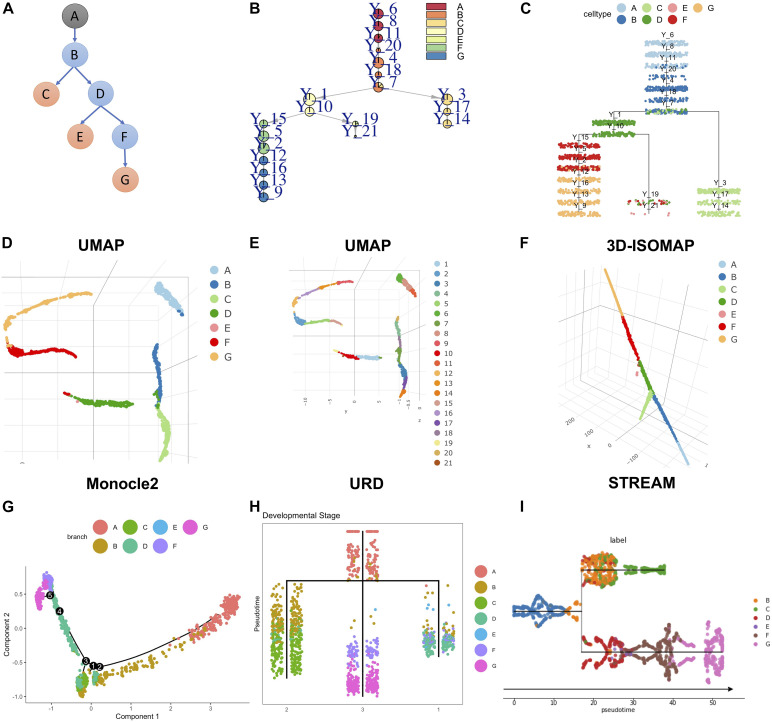
Rare cell branch detection using LISA2 on simulated data. **(A)** The cartoon plot of the trajectory of simulated data with rare cell type E (15 cells, 0.78%). The gray A node is the root. The orange nodes are the leaf branches. The blue nodes are the middle branches. **(B)** Pie tree plot shows the cell types and trajectory. **(C)** Cell cluster tree plot shows the differentiation trajectory. **(D)** The cell type visualization in UMAP. **(E)** The clustering results in UMAP. **(F)** Cell type visualization in 3D L-ISOMAP. **(G)** Monocle 2 trajectory for the simulated data (top 10 PCs used). **(H)** The URD trajectory for the simulated data. **(I)** The STREAM trajectory for the simulated data (all genes were used).

To test the ability of LISA2 to derive rare cell branches, we simulated a dataset by using PROSSTT (Papadopoulos et al., 2019). The simulated development branch is shown in Figure 4A. There are seven cell types marked from A to G. The numbers of cells are 300 in cell types A–D, 350 in cell types F and G, and 15 in the rare cell type E (0.78% of the total number of cells). Figures 4B,C show the trajectory built by LISA2 and Figure 4F shows the 3D L-ISOMAP visualization. We used *k* = 4 for kNN graph construction and clustering using the Leiden algorithm. The smaller the *k* is, the more clusters it produces. Hence, a smaller *k* would help identify rare cell types. The clustering results annotated by cell labels and cluster IDs are shown in the UMAP space (Figures 4D,E). We find that clusters 19 (10, 0.52%) and 21 (5, 0.26%) contain cells from E. In LISA2, cluster 6 is set as root. Clusters 19 and 21 are set as sub leaves group with a linear mode. Cluster 14 is set as sub leaves group. Cluster 9 is set as sub leaves group. Figures 4G–I show the trajectories built by Monocle 2, URD, and STREAM. Compared to Figure 4A, Monocle 2 derived 5 bifurcations and 11 branches with 10 principal components. We also tested 2–10 principal components in Monocle 2, but none of them are consistent with the simulated trajectory. URD and STREAM derived one bifurcation and three branches. In URD, we selected the kNN size as eight because the clustering contains all cells in E. For STREAM, we also adjusted the number of principal components, neighbors, and clusters. In Figure 4I, we set three principal components, 15 neighbors, and 20 clusters. Only LISA2 derived the same bifurcation and branches as those in Figure 4A. The LISA2 trajectories show clearer cell types along the branches than URD. Hence, LISA2 can detect a rare cell type branch with proper clustering. In LISA2, we can adjust the neighbor size in the kNN graph for clustering. Users may also use other rare cell type clustering methods to replace the clustering algorithm in LISA2.

### Comparing LISA2 With Other Methods

**FIGURE 5:**
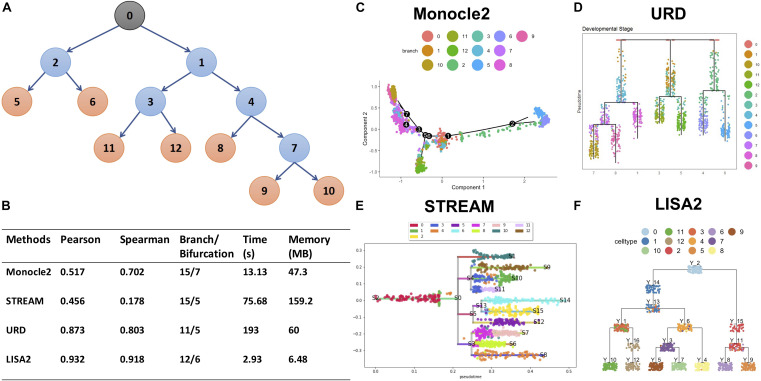
Comparisons of LISA2 with other methods on simulation data. **(A)** The cartoon plot of the trajectory of simulation data with raw branches. The black 0 node is the root. The orange nodes are the leaf branches. The blue nodes are the middle branches. **(B)** Comparisons of the trajectories from four methods on pseudotime, branch, running time, and memory usage. **(C)** The Monocle 2 trajectory for the simulation data (top 20 PCs used). **(D)** The URD trajectory for the simulation data (38 cells not visited). **(E)** The STREAM trajectory for the simulation data (all genes were used). **(F)** The LISA2 trajectory for the simulation data.

We used the simulated dataset with six bifurcations obtained from PROSSTT (Papadopoulos et al., 2019) to compare LISA2 with Monocle 2, URD, and STREAM. The simulated data contain 1,300 cells and 500 genes. Its trajectory includes 7 leaves and 13 branches (Figure 5A). The simulated counts were normalized by the library size in each cell. The scaling factor for each cell was simulated from a log-normal distribution with mean 0 and scale 0.7. For Monocle 2, we used the raw count data as the input. The top 20 PCs were used for DDRTree. Monocle 2 produced 15 branches and 7 bifurcations (Figures 5B,C). When the number of PCs decreased, the numbers of the branches and bifurcations also decreased, and *vice versa*. Monocle 2 could not distinguish branch 11 and 12 and produced more bifurcations. For URD, the raw read counts were normalized internally, and 20 PCs were used for dimension reduction. URD resulted in 11 branches and 5 bifurcations, which separated the leaf branches while missing some internal nodes (Figures 5B,D). The running time of URD was the highest among the four methods. For STREAM, the normalized data from PROSSTT were logged and treated as the input. All genes were used in STREAM, and 20 PCs were used for dimension reduction. STREAM produced more branches compared to the trajectory of the simulation data. Furthermore, branch 1 was regarded as a leaf node in STREAM (Figure 5E). For LISA2, the normalized and logged data were used as the input. We selected 10 PCs for UMAP visualization and performed clustering using 12 neighbors. Clusters 9, 8, 4, 7, 5, 12, and 10 were used as the leaf nodes. The LISA2 trajectory has 6 bifurcations and 12 branches, which were most consistent with the simulated trajectory among the four methods being compared (Figure 5F). The correlation coefficients between the simulated and estimated pseudotime also suggest that LISA2 has the highest performance in pseudotime estimation. Moreover, the running time and memory usage of LISA2 are the lowest among all the four methods being compared (Figure 5B).

We also compared the trajectories obtained from LISA2 with those obtained from Monocle 2, STREAM, and URD on the cerebellum, diencephalon, and HSC datasets (Supplementary Figure 8). For STREAM, it could not work with the cerebellum and diencephalon datasets because of memory exhaustion error. We used the same input data and PCs for the latter three methods and tried to adjust their parameters to obtain optimal results. URD could not directly process the scaled scATAC-seq data. Only a small proportion of cells are walked in the URD, and the trajectory failed to build. For URD, the cerebellum trajectory was not as good as that of LISA2. Some cells were not walked in the URD trajectory even though we adjusted its parameters for the cerebellum (Supplementary Figure 8C). The diencephalon trajectory can be visualized in Guo and Li (2019), and LISA2 shows the same performance as URD. For Monocle 2, the results were largely determined by the PCs used. The more PCs were used, the more branches it produced. Monocle 2 could not work well for convergent and parallel trajectories such as those in the cerebellum and diencephalon datasets. It also derived too many branches from the scATAC-seq HSC data (Supplementary Figures 8A,B,D). STREAM worked well for the HSC data (Chen H. et al., 2019), while costing a large memory and a long time to compute the MLLE. STREAM could not work on the diencephalon and cerebellum datasets due to memory issue, although the cell numbers are below 10,000. Overall, unlike URD and LISA2, Monocle 2 and STREAM did not specify root and leaves, which limited their performance on convergent or parallel branches; URD required a long time to run on large datasets and it was complex for users to adjust the parameters.

At last, we tested how the *k* size in kNN graph affects the clustering and trajectory results on the simulation dataset. In Supplementary Figure 9, we show the clustering visualization in UMAP with *k* from 4 to 20. As *k* increases, the number of clusters decreases overall. From the cell trajectory shown in Supplementary Figure 10, we found that from *k* = 4 to *k* = 12 in kNN graph, the trajectory is consistent with the known trajectory. The higher the number of clusters is, the more complex the trajectory will be. From *k* = 14–20, cell type 8 (marked as yellow cells in Supplementary Figure 10) is directly connected to cell type 7, which is not consistent with the known trajectory. Hence, if *k* is too large, the number of clusters will be small, and some small branches may disappear. Hence, users can set a smaller *k* to discover finer branches.

## Discussion

We developed LISA2 for single-cell trajectory analysis with user-defined root and leaf clusters. By applying LISA2 to two simulated datasets, two real scRNA-seq datasets and one scATAC-seq dataset, we have illustrated the versatility of LISA2 to reconstruct complex trajectories based on single-cell transcriptomics and epigenomics data. With the learned trajectory, we applied PTA to analyze each branch and discovered main gene expression trends and the corresponding genes. Using the known markers and transcription factors, we validated the ability of PTA to discover important gene patterns along the branches. In PTA, we used the average gene expression values of neighboring cells to reduce noise and running time.

LISA2 was designed as a fast, flexible, and scalable method. LISA2 can do PCA for gene expression matrix with # genes × cells <10^10^, as suggested previously (Tsuyuzaki et al., 2020). It adopted fast and scalable graph-based community detection (Leiden and Louvain) algorithms (Blondel et al., 2008; Traag et al., 2019), which were also integrated in Seurat’s pipeline. Hence, LISA2 can be used as a stand-alone platform or downstream of Seurat for trajectory analysis. In trajectory analysis, LISA2 provides a flexible way to group the leaf clusters by linear, parallel, or tree structures. As the trajectory reconstruction is based on clusters, it is very fast to run a spanning tree with specified root and leaves. In addition, users can also run MST without any prior information for exploratory analysis in LISA2. For single-cell trajectory visualization, we used fast L-ISOMAP to view the cell differentiation in 3D space. The trajectory can be added in the 3D L-ISOMAP by connecting the cluster centers.

We compared LISA2 with Monocle 2, STREAM and URD. Monocle 2 and STREAM can build tree structures without known root or end nodes. They were designed to build divergent tree structures. However, for parallel or convergent tree structures, they may produce wrong branches. Users can adjust the number of principal components in Monocle 2 and multiple parameters in STREAM to explore the trajectory. They are both limited by memory problems when running large datasets. URD is designed to solve the problem of complex tree trajectory for the zebrafish dataset initially. By specifying the root and leaf clusters, URD works well for the zebrafish dataset. But zebrafish embryo differentiation cannot be fully described by tree structures due to converging/diverging behaviors (Wagner et al., 2018; Tritschler et al., 2019). In addition, adjusting parameters is also complex in URD. Compared to other methods, LISA2 is flexible and easy to use. The running time of LISA2 is also shorter compared to URD, Monocle 2, and STREAM.

There are also some limitations in scRNA-seq such as the technical/biological noise and sparsity in gene expression. Moreover, cells are sampled from the tissue with a “snapshot” approach. But cell states are dynamic. Branch points in the trajectory may be hypothetical and lagged behind the real cell fate decision (Tritschler et al., 2019; Lähnemann et al., 2020; Savulescu et al., 2020; Wagner and Klein, 2020). These factors may disturb feature selection, cell clustering, and visualization. We have tested LISA2 in non-divergent datasets (cerebellum and diencephalon) and simulated a rare cell type dataset. It suggests that our method has the potential for asynchronous or irregular differentiation studies (Beck and Blanpain, 2013; Kotton and Morrisey, 2014; Tritschler et al., 2019). LISA2 is in principle not limited to scRNA-seq and scATAC-seq. It has the potential for modeling other types of data. It may also be adapted to integrate multiple types of data. LISA2 is currently used for linear, parallel or tree trajectory learning. In the future, it can be extended for loop structures such as cell cycle.

## Materials and Methods

### Dimension Reduction

Raw data can be processed by LISA2 to filter low-quality cells and keep highly variable genes. Users can also employ other tools such as Seurat to preprocess the scRNA-seq data. Then, the filtered data can be processed by PCA, UMAP, and L-ISOMAP.

After PCA, we select top ranked PCs for UMAP and L-ISOMAP. Then, we use the selected PCs to do UMAP. Users can also adjust the number of PCs to acquire a reasonable UMAP visualization.

### Graph-Based Clustering

The clustering method is based on the community detection. We built a kNN graph based on the selected PCs. Then, we use a community detection (Leiden or Louvain) algorithm (Blondel et al., 2008; Traag et al., 2019) to cluster the cells. Because the clustering results depend on the kNN graph, we set a range of *k*-values (from 4 to 20) and obtained corresponding clusters. Users should determine which clusters are better for their applications. The community detection methods are often fast. The Leiden algorithm improves the graph connectivity problem in communities and runs faster than Louvain. In addition, users can also apply the clustering algorithms in other methods such as LISA (Chen Y. et al., 2019) and LrSClust (Wu et al., 2021).

### 3D L-ISOMAP

After clustering, we calculate the graph hubs in the corresponding kNN graph, which has the most number of connections with other nodes in each cluster. The L-ISOMAP in LISA2 uses these graph hubs as landmark points, which is similar to LISA. Compared to LISA, LISA2 uses 3D L-ISOMAP to visualize the complex development process. We modified the source code of the dimRed package and improved the running time of L-ISOMAP. By modifying the order of computing the shortest distance between landmark cells and the other cells, the running time of L-ISOMAP decreases significantly compared to the dimRed package (Supplementary Table 1). We also implemented parallelization in L-ISOMAP for datasets with a large number of cells.

### Building the Spanning Tree With Specified Root and Leaves

With the clusters, we designed a spanning tree with specified root and leaf nodes. The MST method can produce the shortest paths to connect all vertexes. However, it may also neglect the real biological development process.

The spanning tree methods are as follows:

Step 1. We calculate the neighbor distance matrix and graph distance matrix for the clusters. In the neighbor distance matrix, if two clusters are connected, we compute the mean distance of the edges. If two clusters are not directly connected, we set the distance as NA. The graph distance matrix is computed for each two clusters based on the shortest path.
Step 2. User specifies the root cluster. Then, LISA2 builds the kNN graph for the non-leaf clusters. The neighbor size is set from 3 until the graph is connected. The leaf clusters are then added to its nearest non-leaf clusters. For the leaf nodes, if one leaf group contains multiple leaf clusters, the user can specify linear, parallel, and MST structures (Figure 1E). For each leaf group, the cluster having the shortest distance to the non-leaf clusters can be selected.
Step 3. For each leaf cluster, we find the shortest path from the root to the leaf cluster. For isolated non-leaf clusters, we find the cluster with the shortest distance to the isolated non-leaf cluster and merge it into the graph. In the final spanning tree, the degree of non-leaf clusters must be at least two. The degree of leaf nodes is only one.

The spanning tree method can produce the tree trajectory for the clusters by user-specified root (e.g., cluster 17) and leaves (e.g., clusters 2, 8, 9, 10, 6) (Figure 1F) for the cerebellum dataset. We can visualize the tree trajectory by L-ISOMAP (Figure 3E), pie tree (Figure 1F), or cluster tree (Figure 1G).

### Pseudotime and Principal Trend Analysis

Based on the trajectory, LISA2 derives the pseudotime for the global branches (Figure 1H). Similar to LISA, LISA2 first maps the cells on the tree. The difference is that LISA2 maps each cell to the edge in which the vertex is the cluster that the cell belongs to.

Principal trend analysis is proposed to discover principal time-course trends from gene expression data (Zhang and Davis, 2013; Zhang and Ouyang, 2018). It also quantifies the contribution of each gene to the trends. Here, we used “score” to represent the contribution of each gene to each trend. In most cases, the score of most genes is zero. Only subsets of genes contribute to the trends. Hence, we sought to use PTA to find the marker genes that may represent the specific branch development. PTA can iteratively identify multiple trends from the time-course gene expression data. We used PTA to generate three trends, named as “rank 1” to “rank 3,” to represent the main gene expression patterns along the branches (Figure 1I).

## Data Availability Statement

Publicly available datasets were analyzed in this study. The LISA2 source code package can be download from GitHub (https://github.com/ouyang-lab/LISA2). The simulated datasets are from https://github.com/soedinglab/prosstt/blob/master/examples/many_branches_cells.ipynb. The cerebellum dataset is from https://github.com/JLiLab/scRNAseq_Cerebellum. The diencephalon dataset is from GSE122012. The scATAC-seq data is from https://www.dropbox.com/sh/zv6z7f3kzrafwmq/AACAlU8akbO_a-JOeJkiWT1za?dl=0.

## Author Contributions

All authors actively contributed to the results and discussions the procedures. YC contributed to data preprocessing, method design, trend analysis, and write-up. YZ contributed to realization of workflows, trend analysis, and write-up. JL contributed to data preprocessing and annotation. ZO contributed to conception of the project, method design, realization of workflows, trend analysis, and write-up.

## Conflict of Interest

The authors declare that the research was conducted in the absence of any commercial or financial relationships that could be construed as a potential conflict of interest.

## Publisher’s Note

All claims expressed in this article are solely those of the authors and do not necessarily represent those of their affiliated organizations, or those of the publisher, the editors and the reviewers. Any product that may be evaluated in this article, or claim that may be made by its manufacturer, is not guaranteed or endorsed by the publisher.

## Supplementary Material

Supplementary Figure 1 The marker gene expression of each branch in cerebellum. The cells are ordered by pseudo time. In each heatmap, each column is corresponding to a cluster in the branch. The legend in the heatmap is corresponding to the clustering results in D. **(A)** Marker genes expression along PC1 branch. **(B)** Marker genes expression along PC2 branch. **(C)** Marker genes expression along PC3 branch. **(D)** Clustering results shown in UMAP. **(E)** Marker genes expression along IsN, lCN branch. **(F)** Marker genes expression along mCN branch. **(G)** Marker genes expression along GC branch. **(H)** Cell types shown in UMAP. **(I)** Marker genes expression along GABIN branch. **(J)** Marker genes expression along BG branch. **(K)** Compare the marker genes expression along BG and GABA.pro branch. **(L)** Pie tree plot shows the trajectory by LISA2. Purkinje cell (PC), GABAergic Interneurons (GABA.IN), Bergermann glia (BG), medial cerebellar nuclei (mCN), Isthmic nuclear neurons (IsN), lateral cerebellar nuclei (lCN), granule cells (GC), GABAergic progenitor (GABA.pro).

Supplementary Figure 2 The main gene expression trends (rank 1: red, rank 2: blue, rank 3: green) in each branch **(A–H)** in cerebellum are shown from rank 1 to rank 3 by PTA analysis. Here we only show the heatmap of normalized gene expression in rank 1. For all branches, we did down sampling using 50 as a group to compute the average signals as input of PTA. The gene expression data is the scaled data from Seurat. In the heatmap **(A–H)**, all genes in rank 1 are ranked by the scores (values in the bar plot) from negative to positive. Negative value means that the gene expression is reverse to the trend. Positive value means that the gene expression is consistent with the trend. We set a threshold 0.03 to filter the genes with low absolute scores and show the genes with top 30 positive and smallest 30 negative scores. Purkinje cell (PC), GABAergic Interneurons (GABA.IN), Bergermann glia (BG), medial cerebellar nuclei (mCN), Isthmic nuclear neurons (IsN), lateral cerebellar nuclei (lCN), granule cells (GC), GABAergic progenitor (GABA.pro).

Supplementary Figure 3 The PTA scores of marker gene expressions of each branch in cerebellum **(A–H)** from rank 1 to 3. In each heatmap, three columns are corresponding to the rank 1 to 3. Purkinje cell (PC), GABAergic Interneurons (GABA.IN), Bergermann glia (BG), medial cerebellar nuclei (mCN), Isthmic nuclear neurons (IsN), lateral cerebellar nuclei (lCN), granule cells (GC), GABAergic progenitor (GABA.pro).

Supplementary Figure 4 The marker gene expression of each branch in diencephalon **(A–F)**. The cells are ordered by pseudo time. In each heatmap, each column is corresponding to a cluster in the branch. The legend in the heatmap is corresponding to the clustering results in Figure 2B. Prethalamic neuros (pTh.N), rostral thalamic neurons (rTh.N), zona limitans intrathalamica (ZLI), epithalamic neuros (ETh.N), rostral pretecal neurons (rPT.N), caudal thalamus neuro I (cTh.N1), caudal thalamus neuro II (cTh.N2).

Supplementary Figure 5 The main gene expression trends (rank 1: red, rank 2: blue, rank 3: green) in each branch **(A–F)** in diencephalon are shown from rank 1 to rank 3 by PTA analysis. Here we only show the heatmap of normalized gene expression in rank 1 **(A–F)**. For all branches, we did down sampling using 50 as a group to compute the average signals as input of PTA. The gene expression data is the scaled data from Seurat. In the heatmap, all genes in rank 1 are ranked by the scores (values in the bar plot) from negative to positive. Negative value means that the gene expression is reverse to the trend. Positive value means that the gene expression is consistent with the trend. We set a threshold 0.03 to filter the genes with low absolute scores and show the genes with top 30 positive and smallest 30 negative scores. Prethalamic neuros (pTh.N), rostral thalamic neurons (rTh.N), zona limitans intrathalamica (ZLI), epithalamic neuros (ETh.N), rostral pretecal neurons (rPT.N), caudal thalamus neuro I (cTh.N1), caudal thalamus neuro II (cTh.N2).

Supplementary Figure 6 The PTA scores of marker gene expressions of each branch in diencephalon **(A–H)** from rank 1 to 3. In each heatmap, three columns are corresponding to the rank 1 to 3. Prethalamic neuros (pTh.N), rostral thalamic neurons (rTh.N), zona limitans intrathalamica (ZLI), epithalamic neuros (ETh.N), rostral pretecal neurons (rPT.N), caudal thalamus neuro I (cTh.N1), caudal thalamus neuro II (cTh.N2).

Supplementary Figure 7 The 7-mer DNA sequence features trends (rank 1: red, rank 2: blue, rank 3: green) in HSC are shown from rank 1 to rank 3 by PTA analysis **(A–D)**. Here we only show the heatmap of DNA sequence features in rank 1. For each branch, we did down sampling using 20 as a group to compute the average signals as input of PTA. All7-mer DNA sequences in rank 1 are ranked by the PTA scores (values in the bar plot) from negative to positive. Negative value means that the *z*-score values are reverse to the trend. Positive value means that the *z*-score values are consistent with the trend. We set a threshold 0.03 to filter the DNA sequences with low absolute PTA scores and show the DNA sequence features with top 30 positive and smallest 30 negative scores. Prethalamic neuros (pTh.N), rostral thalamic neurons (rTh.N), zona limitans intrathalamica (ZLI), epithalamic neuros (ETh.N), rostral pretecal neurons (rPT.N), caudal thalamus neuro I (cTh.N1), caudal thalamus neuro II (cTh.N2).

Supplementary Figure 8 Compare the trajectories of URD, Monocle2 and STREAM on the cerebellum, diencephalon, and HSC datasets. **(A)** Cerebellum trajectory built by Monocle2. **(B)** Diencephalon trajectory built by Monocle2. **(C)** Cerebellum trajectory built by URD. **(D)** HSC trajectory built by Monocle2 based on the *z*-scored scATAC-seq data. STREAM cannot work on the cerebellum and diencephalon datasets. The HSC trajectory from STREAM can be found in Chen H. et al. (2019). URD cannot work for the *z*-scored scATAC-seq dataset of HSC. The diencephalon trajectory built by URD can be found in Guo and Li (2019).

Supplementary Figure 9 Clustering visualization in UMAP on simulated dataset with different *k* size in kNN graph. As the *k* increases, the number of clusters decreases.

Supplementary Figure 10 Trajectory estimation for the simulated dataset using different *k* in kNN graph. The clustering was obtained by constructing the kNN graph followed by Leiden community detection. Then we applied LISA2 to obtain the final trajectory. The number of clusters decreases as the *k* size increase.

Supplementary Table 1 Compare running time (seconds) of L-ISOMAP in LISA and LISA2. The computer configurations are 64-bit Windows 8.1, Intel(R) Core i5-4210M CPU, and 8GB memory. Results were obtained using the same computer configuration.
