# Supplementary material for: LISA2: Learning Complex Single-Cell Trajectory and Expression Trends
Source: Front Genet. 2021 Aug 23;12:681206. doi: 10.3389/fgene.2021.681206 (PMC8428276; doi:10.3389/fgene.2021.681206)
Supplement: Supplementary file 1 [file Data_Sheet_1.zip › data sheet 1/Suppl Data_diencephalon.html]

diencephalon


# diencephalon

### 3D-ISOMAP
