# Supplementary material for: LISA2: Learning Complex Single-Cell Trajectory and Expression Trends
Source: Front Genet. 2021 Aug 23;12:681206. doi: 10.3389/fgene.2021.681206 (PMC8428276; doi:10.3389/fgene.2021.681206)
Supplement: Supplementary Figure 1 — The marker gene expression of each branch in cerebellum. The cells are ordered by pseudo time. In each heatmap, each column is corresponding to a cluster in the branch. The legend in the heatmap is corresponding to the clustering results in D. (A) Marker genes expression along PC1 branch. (B) Marker genes expression along PC2 branch. (C) Marker genes expression along PC3 branch. (D) Clustering results shown in UMAP. (E) Marker genes expression along IsN, lCN branch. (F) Marker genes expression along mCN branch. (G) Marker genes expression along GC branch. (H) Cell types shown in UMAP. (I) Marker genes expression along GABIN branch. (J) Marker genes expression along BG branch. (K) Compare the marker genes expression along BG and GABA.pro branch. (L) Pie tree plot shows the trajectory by LISA2. Purkinje cell (PC), GABAergic Interneurons (GABA.IN), Bergermann glia (BG), medial cerebellar nuclei (mCN), Isthmic nuclear neurons (IsN), lateral cerebellar nuclei (lCN), granule cells (GC), GABAergic progenitor (GABA.pro). [file Data_Sheet_1.docx]

Supplementary Material

# Supplementary Figures and Tables

## Supplementary Figures


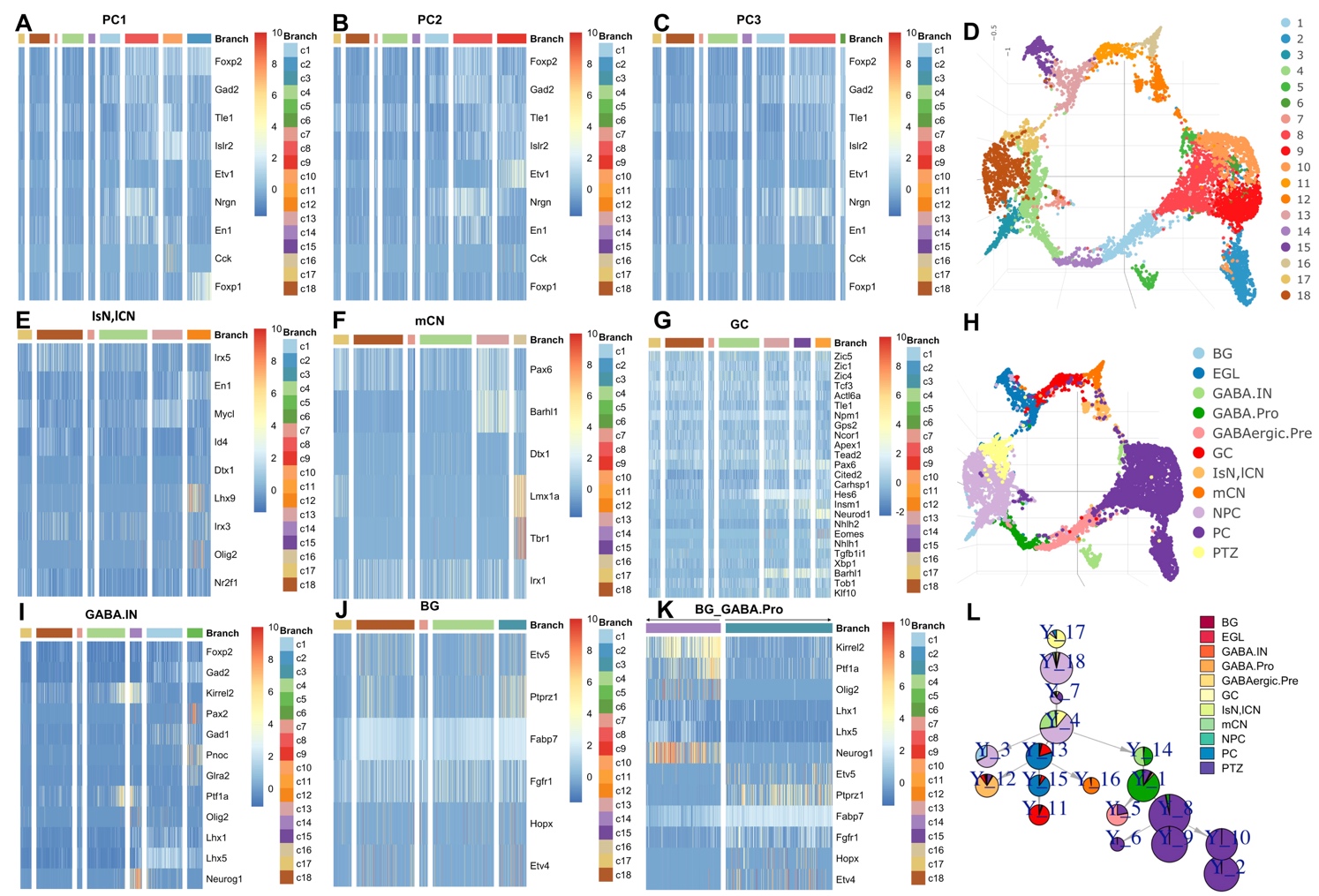


**Supplementary Figure 1.** The marker gene expression of each branch in cerebellum. The cells are ordered by pseudo time. In each heatmap, each column is corresponding to a cluster in the branch. The legend in the heatmap is corresponding to the clustering results in D. A. Marker genes expression along PC1 branch. B. Marker genes expression along PC2 branch. C. Marker genes expression along PC3 branch. D. Clustering results shown in UMAP. E. Marker genes expression along IsN, lCN branch. F. Marker genes expression along mCN branch. G. Marker genes expression along GC branch. H. Cell types shown in UMAP. I. Marker genes expression along GABIN branch. J. Marker genes expression along BG branch. K. Compare the marker genes expression along BG and GABA.pro branch. L. Pie tree plot shows the trajectory by LISA2. Purkinje cell (PC), GABAergic Interneurons (GABA.IN), Bergermann glia (BG), medial cerebellar nuclei (mCN), Isthmic nuclear neurons (IsN), lateral cerebellar nuclei (lCN), granule cells (GC), GABAergic progenitor (GABA.pro).

**
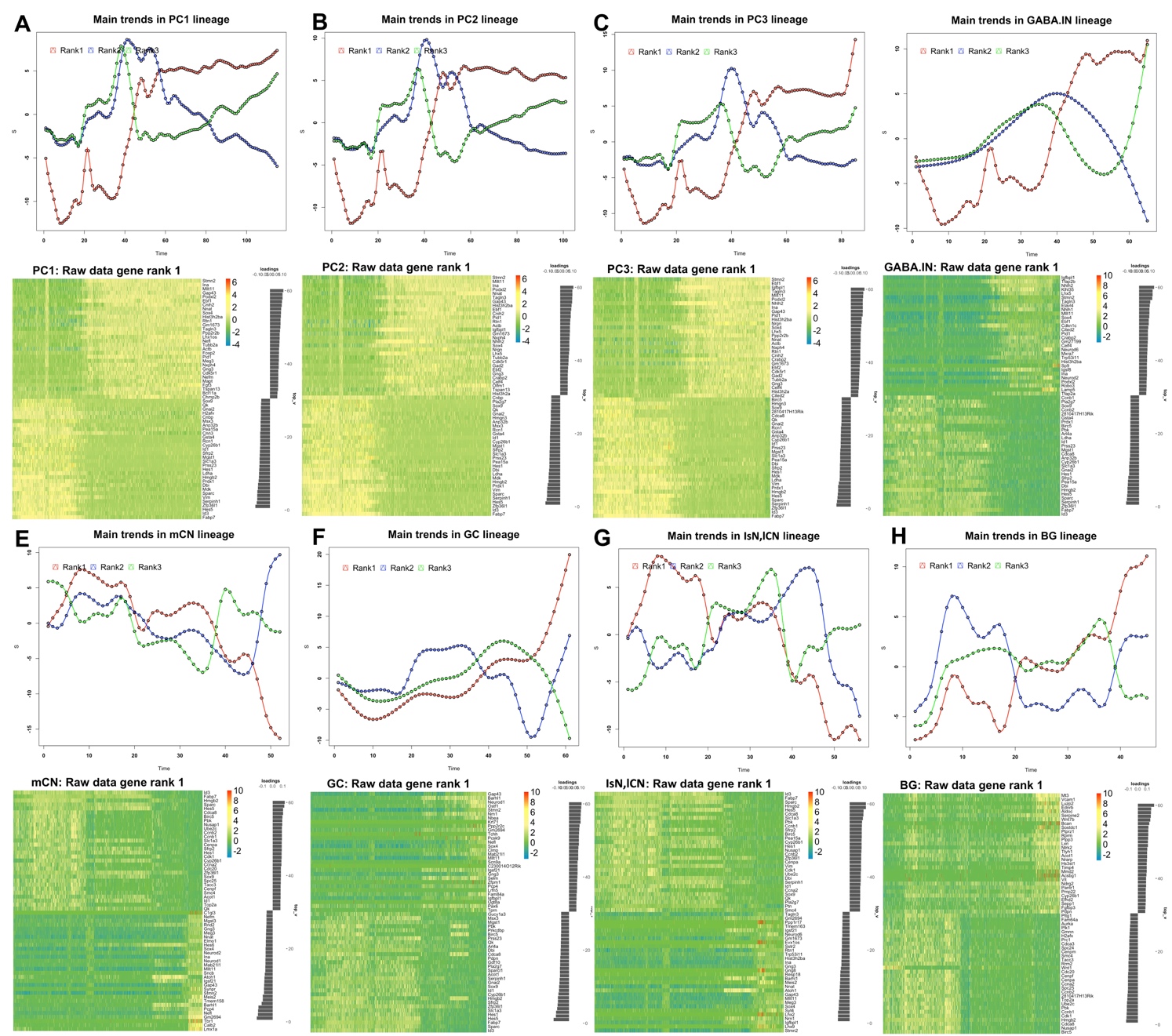
**

**Supplementary Figure 2.** The main gene expression trends (rank 1: red, rank 2: blue, rank 3: green) in each branch (A-H) in cerebellum are shown from rank 1 to rank 3 by PTA analysis. Here we only show the heatmap of normalized gene expression in rank 1. For all branches, we did down sampling using 50 as a group to compute the average signals as input of PTA. The gene expression data is the scaled data from Seurat. In the heatmap A-H, all genes in rank 1 are ranked by the scores (values in the bar plot) from negative to positive. Negative value means that the gene expression is reverse to the trend. Positive value means that the gene expression is consistent with the trend. We set a threshold 0.03 to filter the genes with low absolute scores and show the genes with top 30 positive and smallest 30 negative scores. Purkinje cell (PC), GABAergic Interneurons (GABA.IN), Bergermann glia (BG), medial cerebellar nuclei (mCN), Isthmic nuclear neurons (IsN), lateral cerebellar nuclei (lCN), granule cells (GC), GABAergic progenitor (GABA.pro).


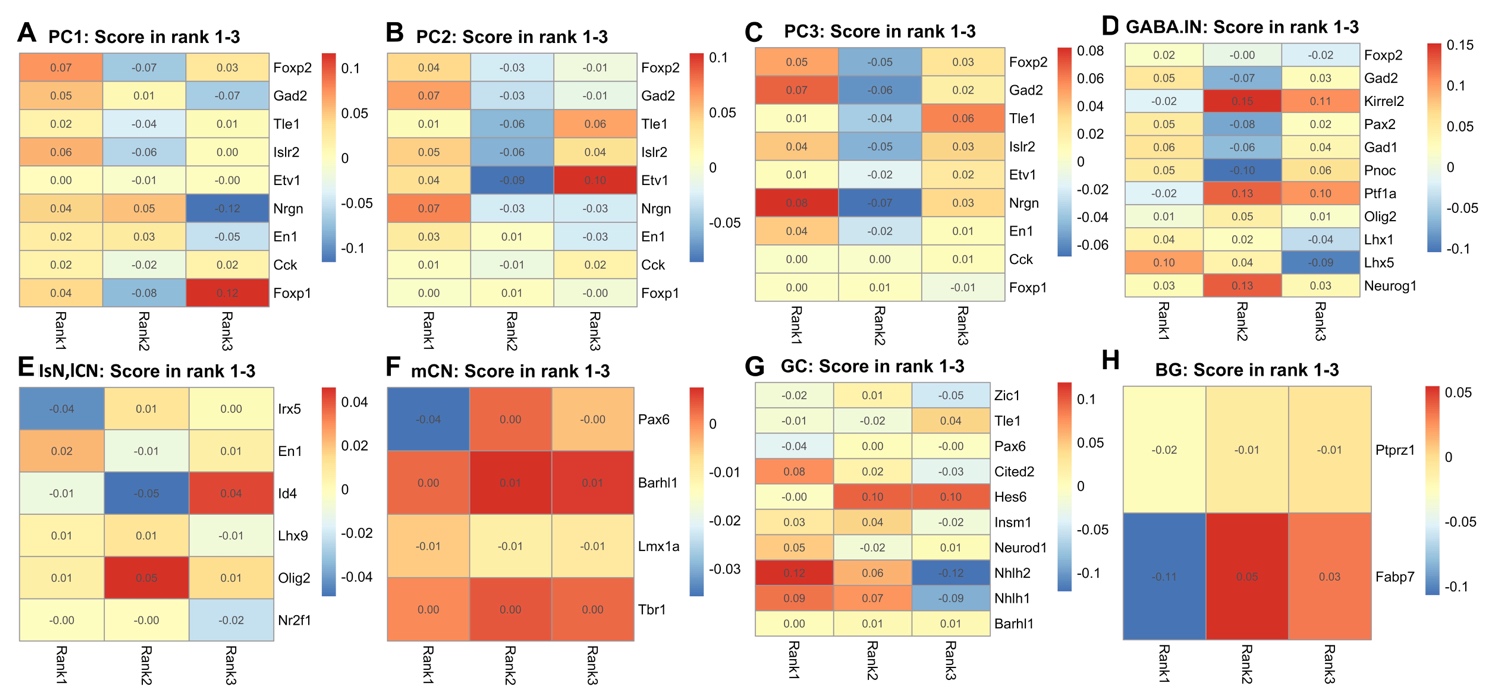


**Supplementary Figure 3.** The PTA scores of marker gene expressions of each branch in cerebellum (A-H) from rank 1 to 3. In each heatmap, three columns are corresponding to the rank 1 to 3. Purkinje cell (PC), GABAergic Interneurons (GABA.IN), Bergermann glia (BG), medial cerebellar nuclei (mCN), Isthmic nuclear neurons (IsN), lateral cerebellar nuclei (lCN), granule cells (GC), GABAergic progenitor (GABA.pro).


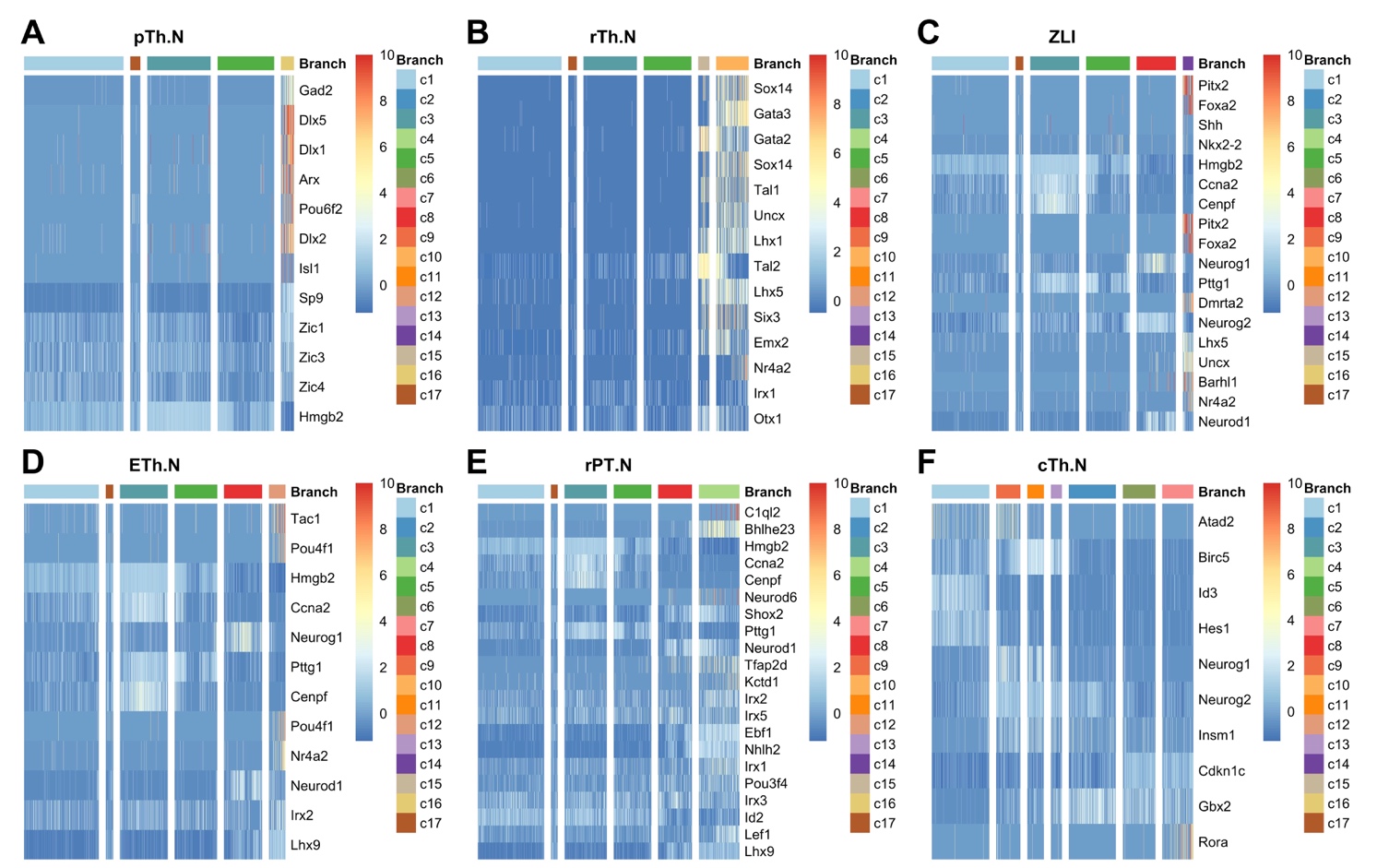


**Supplementary Figure 4.** The marker gene expression of each branch in diencephalon (A-F). The cells are ordered by pseudo time. In each heatmap, each column is corresponding to a cluster in the branch. The legend in the heatmap is corresponding to the clustering results in Fig.2-B. Prethalamic neuros (pTh.N), rostral thalamic neurons (rTh.N), zona limitans intrathalamica (ZLI), epithalamic neuros (ETh.N), rostral pretecal neurons (rPT.N), caudal thalamus neuro I (cTh.N1), caudal thalamus neuro II (cTh.N2).


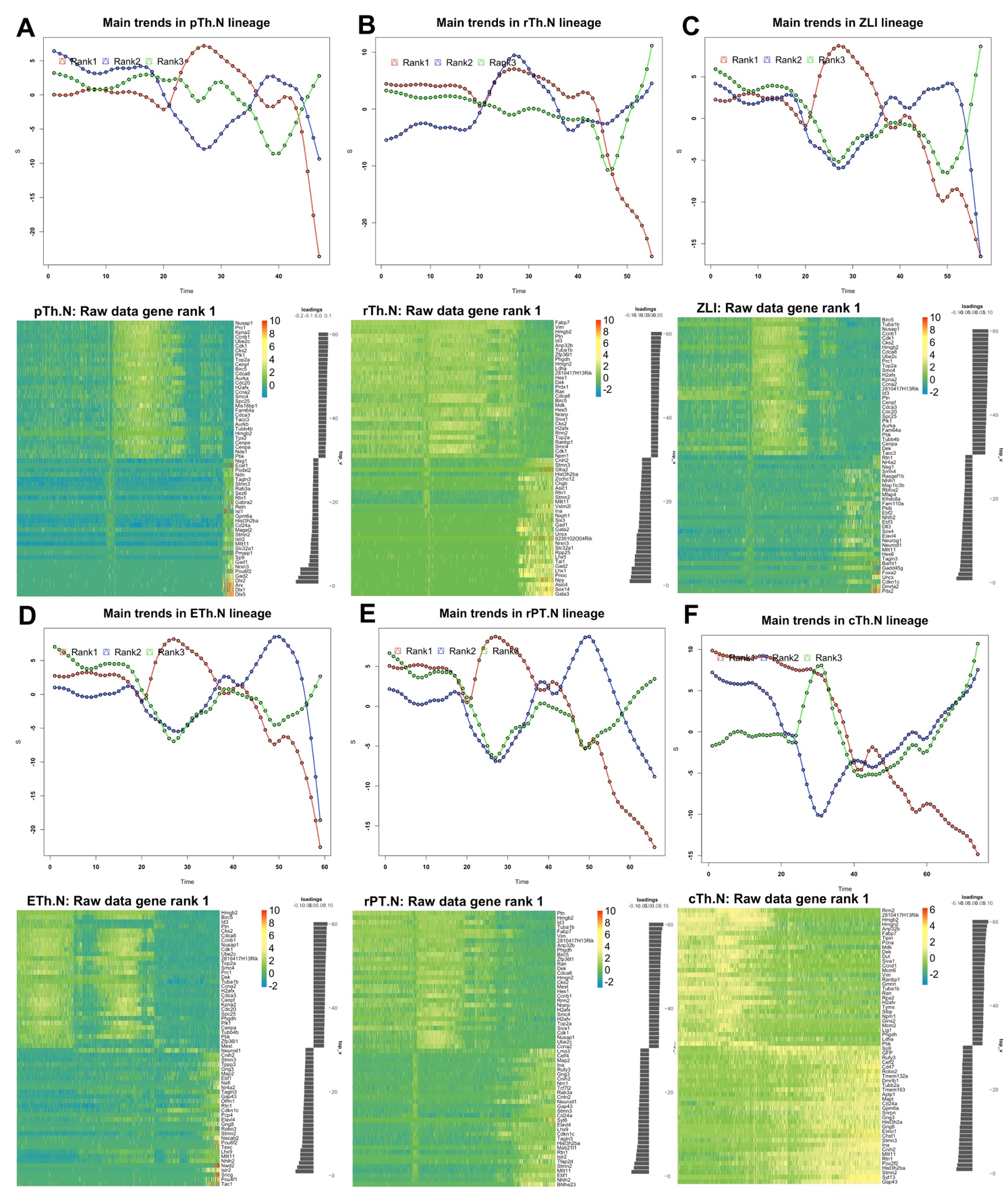


**Supplementary Figure 5.** The main gene expression trends (rank 1: red, rank 2: blue, rank 3: green) in each branch (A-F) in diencephalon are shown from rank 1 to rank 3 by PTA analysis. Here we only show the heatmap of normalized gene expression in rank 1 (A-F). For all branches, we did down sampling using 50 as a group to compute the average signals as input of PTA. The gene expression data is the scaled data from Seurat. In the heatmap, all genes in rank 1 are ranked by the scores (values in the bar plot) from negative to positive. Negative value means that the gene expression is reverse to the trend. Positive value means that the gene expression is consistent with the trend. We set a threshold 0.03 to filter the genes with low absolute scores and show the genes with top 30 positive and smallest 30 negative scores. Prethalamic neuros (pTh.N), rostral thalamic neurons (rTh.N), zona limitans intrathalamica (ZLI), epithalamic neuros (ETh.N), rostral pretecal neurons (rPT.N), caudal thalamus neuro I (cTh.N1), caudal thalamus neuro II (cTh.N2).


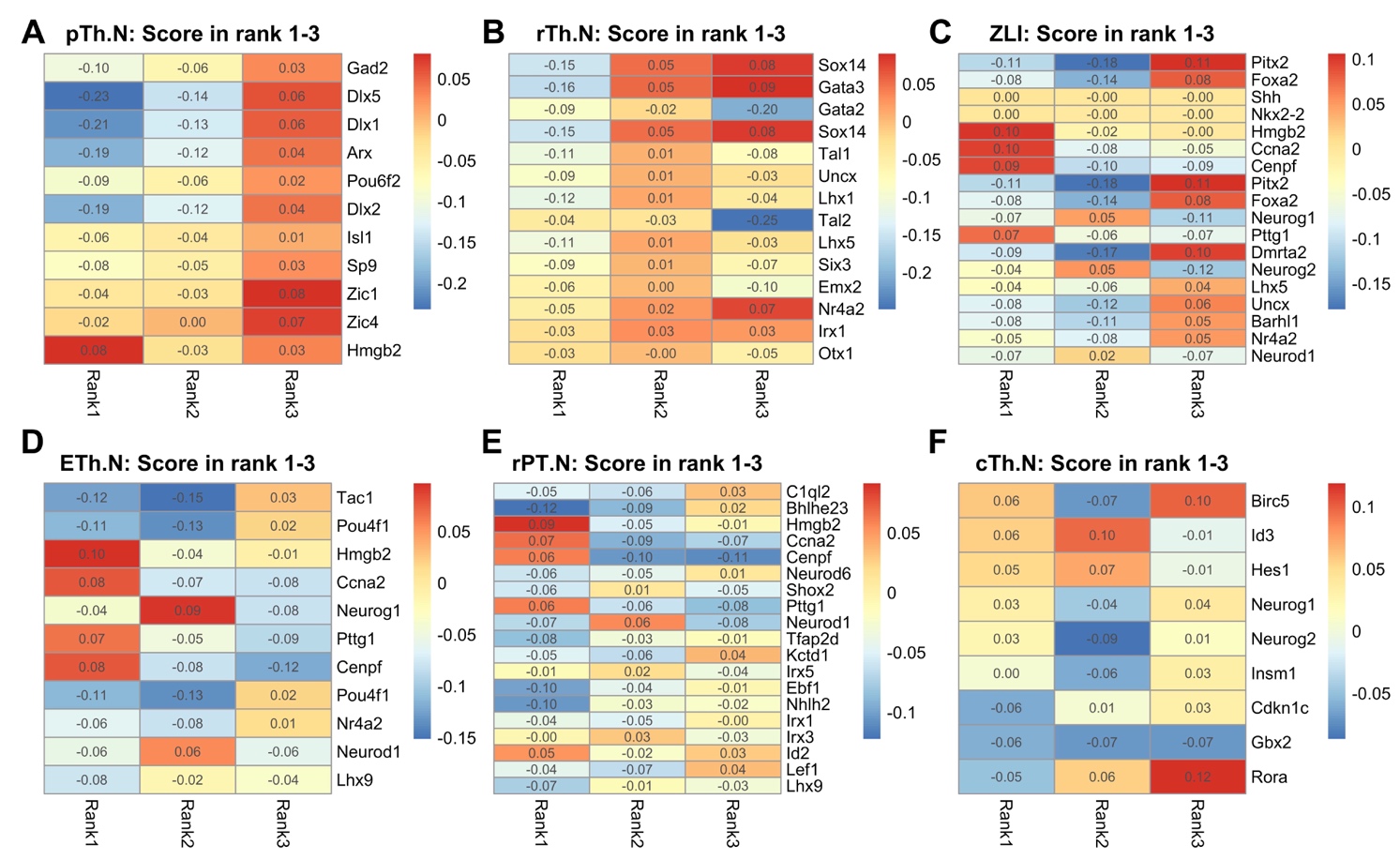


**Supplementary Figure 6.** The PTA scores of marker gene expressions of each branch in diencephalon (A-H) from rank 1 to 3. In each heatmap, three columns are corresponding to the rank 1 to 3. Prethalamic neuros (pTh.N), rostral thalamic neurons (rTh.N), zona limitans intrathalamica (ZLI), epithalamic neuros (ETh.N), rostral pretecal neurons (rPT.N), caudal thalamus neuro I (cTh.N1), caudal thalamus neuro II (cTh.N2).


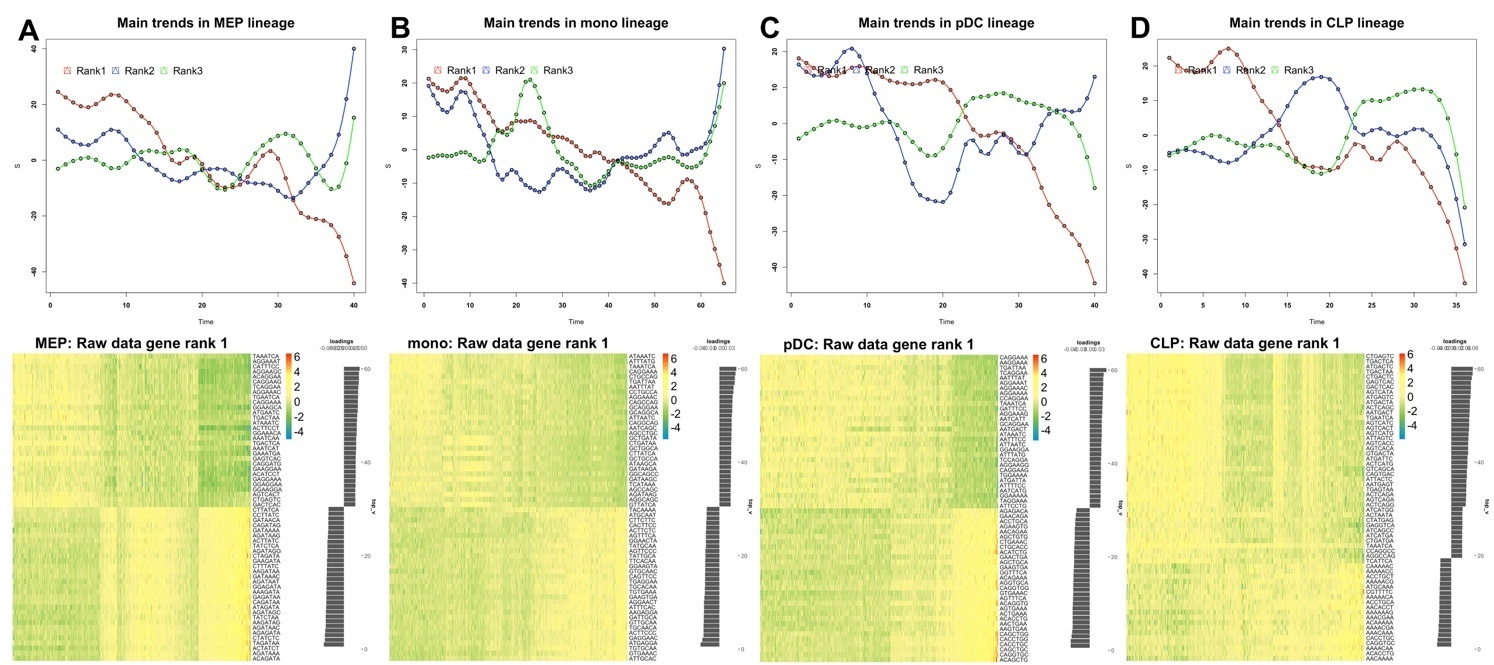


**Supplementary Figure 7.** The 7-mer DNA sequence features trends (rank 1: red, rank 2: blue, rank 3: green) in HSC are shown from rank 1 to rank 3 by PTA analysis(A-D). Here we only show the heatmap of DNA sequence features in rank 1. For each branch, we did down sampling using 20 as a group to compute the average signals as input of PTA. All7-mer DNA sequences in rank 1 are ranked by the PTA scores (values in the bar plot) from negative to positive. Negative value means that the z-score values are reverse to the trend. Positive value means that the z-score values are consistent with the trend. We set a threshold 0.03 to filter the DNA sequences with low absolute PTA scores and show the DNA sequence features with top 30 positive and smallest 30 negative scores. Prethalamic neuros (pTh.N), rostral thalamic neurons (rTh.N), zona limitans intrathalamica (ZLI), epithalamic neuros (ETh.N), rostral pretecal neurons (rPT.N), caudal thalamus neuro I (cTh.N1), caudal thalamus neuro II (cTh.N2).


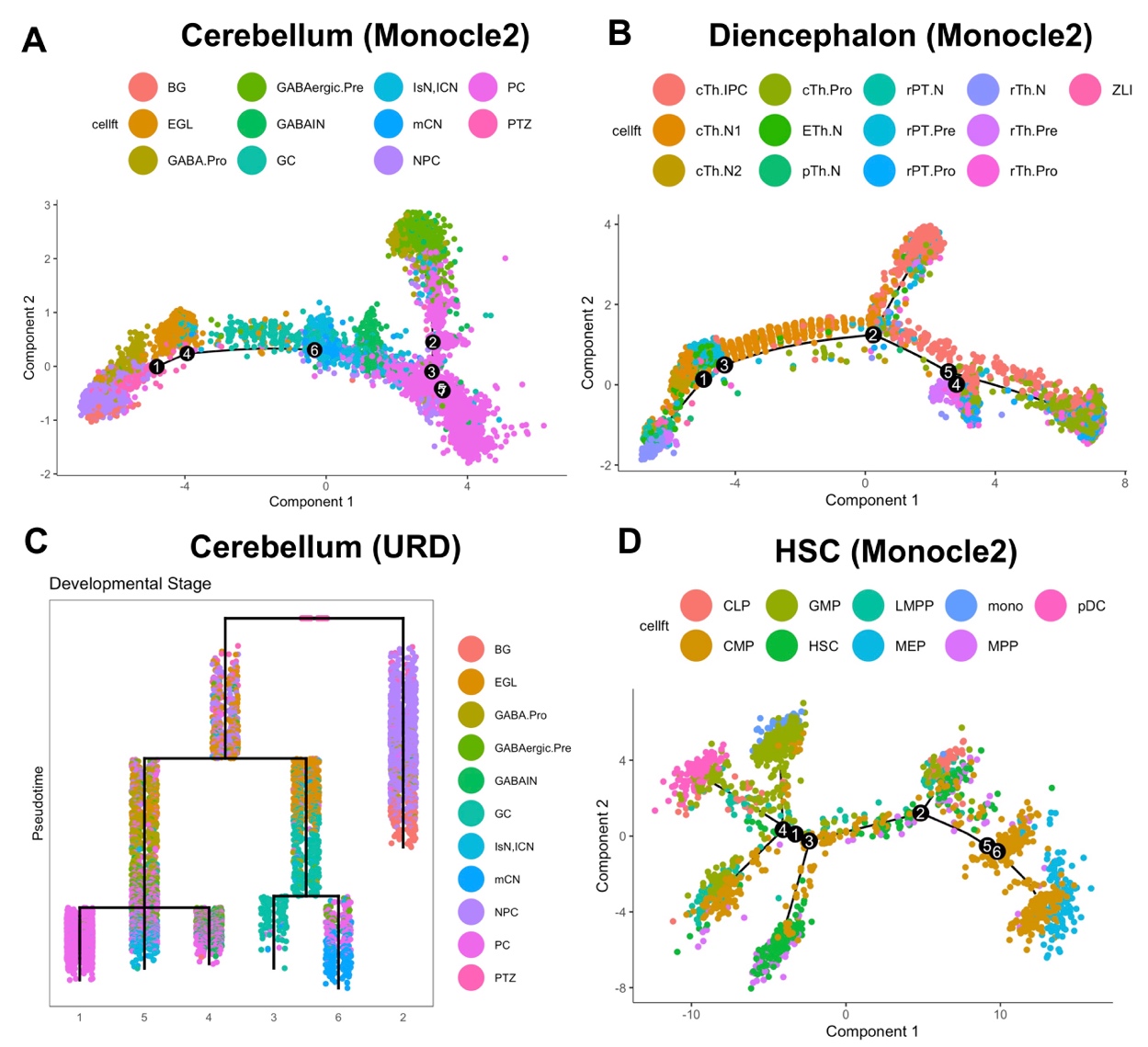


**Supplementary Figure 8.** Compare the trajectories of URD, Monocle2 and STREAM on the cerebellum, diencephalon, and HSC datasets. A. Cerebellum trajectory built by Monocle2. B. Diencephalon trajectory built by Monocle2. C. Cerebellum trajectory built by URD. D. HSC trajectory built by Monocle2 based on the z-scored scATAC-seq data. STREAM cannot work on the cerebellum and diencephalon datasets. The HSC trajectory from STREAM can be found in Chen H. et al., 2019. URD cannot work for the z-scored scATAC-seq dataset of HSC. The diencephalon trajectory built by URD can be found in Guo et al., 2019.


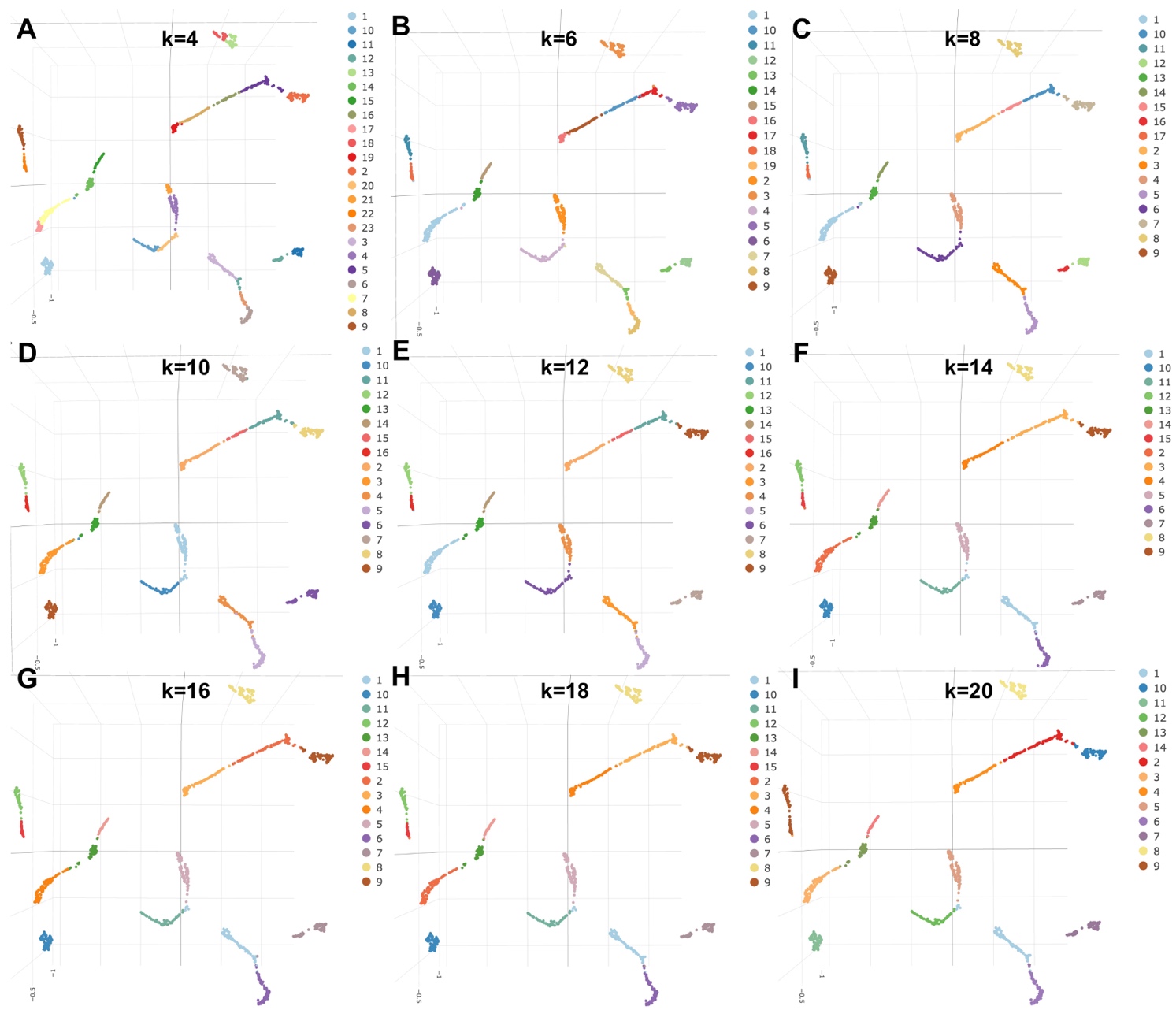


**Supplementary Figure 9.** Clustering visualization in UMAP on simulated dataset with different *k* size in kNN graph. As the *k* increases, the number of clusters decreases.


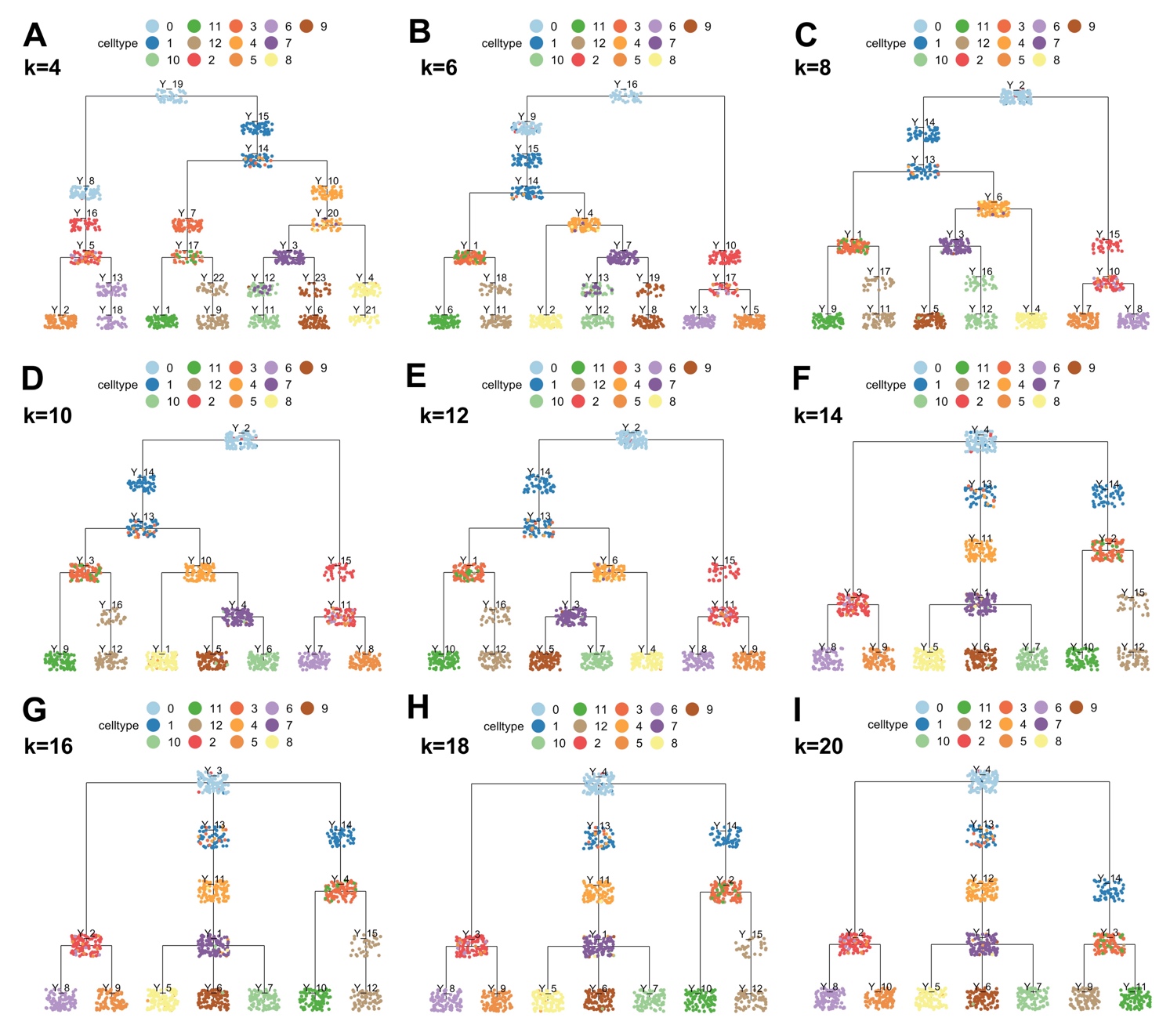


**Supplementary Figure 10.** Trajectory estimation for the simulated dataset using different *k* in kNN graph. The clustering was obtained by constructing the kNN graph followed by Leiden community detection. Then we applied LISA2 to obtain the final trajectory. The number of clusters decreases as the *k* size increase.

## Supplementary Tables

**Supplementary Table1.**Compare running time (seconds) of L-ISOMAP in LISA and LISA2. The computer configurations are 64-bit Windows 8.1, Intel(R) Core i5-4210M CPU, and 8GB memory. Results were obtained using the same computer configuration.

| **Dataset** | **Cell number** | **LISA** | **LISA2** |
| --- | --- | --- | --- |
| Simulation | 1,100 | 2.04 | 0.28 |
| HSC | 2,034 | 9.87 | 0.32 |
| Diencephalon | 6,952 | 244.26 | 1.14 |
| Cerebellum | 9,165 | 591.85 | 21.29 |
